# Automated Control of Surface Defects on Ceramic Tiles Using 3D Image Analysis

**DOI:** 10.3390/ma13051250

**Published:** 2020-03-10

**Authors:** Andrzej Sioma

**Affiliations:** Faculty of Mechanical Engineering and Robotics, Department of Process Control, AGH University of Science and Technology, 30-059 Krakow, Poland; andrzej.sioma@agh.edu.pl; Tel.: +48-12-6175005

**Keywords:** surface defects of ceramic tiles, 3D vision system, 3D image, 3D image analysis

## Abstract

This paper presents a method of acquisition and analysis of three-dimensional images in the task of automatic location and evaluation of defects on the surface of ceramic tiles. It presents a brief description of selected defects appearing on the surface of tiles, along with the analysis of their formation. The paper includes the presentation of the method of constructing a 3D image of the tile surface using the Laser Triangulation Method (LTM), along with the surface imaging parameters employed in the research. The algorithms of three-dimensional surface image analysis of ceramic tiles used in the process of image filtering and defect identification are presented. For selected defects, the method of measuring defect parameters and the method of visualization of defects on the surface are also presented. The developed method was tested on defective products to confirm its effectiveness in the field of quick defect detection in automated control systems installed on production lines.

## 1. Introduction

The increase in the capacity of production lines producing ceramic tiles imposes the introduction of innovative solutions in the field of fault diagnosis and detection systems on product surfaces. In many manufacturing enterprises, the quality inspection of ceramic tiles is carried out by a person supported by sensor or vision systems. Firat et al. presented the study of the description of employee fatigue and the possibility of errors made by them in the implementation of visual assessment of the product related to work time or type of product [1].

Introduction of vision systems to the production lines made it possible to acquire an image and analyse this image in order to identify and locate defects. As part of the vision methods described in the literature, the analyses of shades of grey or colours are usually used. Hanzaeia et al. presented a system based on 2D image analysis with statistical methods to detect the edges of such surface defects as cracks and scratches [2]. Hocenski et al. presented a computer system (CVS) that enables the analysis of the image of tiles and the assessment of defects on their surface using a program written in C ++ with OpenCV libraries used to analyse 2D images [3]. Job Samarawickrama et al. presented a system based on image analysis implemented in the Matlab software that also uses two-dimensional image analysis, both in monochrome and colour [4]. Vaideliene et al. proposed the use of wavelet analysis for the location of defect areas [5]. The authors presented an effective defect detection system on texture images visible on the surface of tiles. In [6], the authors described a methodology for automatically identifying granite varieties. They used spectral information captured by a spectrophotometer and machine learning techniques.

In article [7], the authors presented hybrid 3D-2D laser scanning system for the structural defects recognition of slate slabs. The paper describes defects in roofing slates, parameters describing their quality and method of those parameter control. In [8,9], the authors present machine learning methods in the task of identifying defective slate slabs and present the differences in the results to the adopted learning method.

In most industrial applications, the image analysis system is responsible for detecting defects on a surface. The task of checking the geometry of the surface in the form of a flatness check is done by means of a sensor system (4–8 sensors); these measure the height in designated areas of the tile surface. Most vision systems used in the ceramic industry can be described as two-dimensional due to the information available in the image and the method of surface imaging. They are most often implemented as additional equipment in technological machines and checking stations, the task of which is the implementation of inter-operational inspection of product parameters. Introduction of automated video stands with extended measuring and inspection capabilities is required, especially in mass production carried out in the “zero defects” system, providing a thorough inspection of all products leaving the production line.

This paper presents the method of acquisition and analysis of 3D images in the inspection tasks of selected parameters of a ceramic tile. The paper presents examples of using 3D images in the implementation of tasks to check defects in geometry and surface defects of the product. The advantage of the method is that the three-dimensional image enables the implementation of a much larger number of measurements and analyses, especially in the field of spatial measurements, complementing and extending the currently used vision methods in the video industry based on two-dimensional image analysis [10,11,12,13,14,15].

The geometric defects occurring most often on the surface of ceramic tiles include defects in the flatness of the tile’s usable surface. These defects are visible in the form of curved corners or edges or in the form of a visible buckling on the surface. Geometric defects also include any damages to edges and corners. A large group of defects are also flaws of usable surfaces visible in the form of surface delamination, scratches or local roughness variations. These defects may be very small in size or may be seen on almost the entire surface of the tile. The following drawing shows a large delamination area, located in the centre of the ceramic tile. Implementation of research and development of a system enabling detection of all the described defects requires implementation of imaging of the entire surface of the tile and preparation of a set of reference algorithms to enable the detection of each type of defects.

The objective of the research described in this paper was to develop and implement a system that allows comprehensive assessment of the quality of ceramic tiles. It results from the need to check the quality of the product’s performance in the scope of checking the geometrical dimensions of the ceramic tile combined with simultaneous checking of the presence and parameters of defects on the surface of ceramic products. For this purpose, a vision system was developed using the LTM method, designed to build a three-dimensional image of the surface of the tile. It was assumed that the video system operation parameters and 3D image analysis algorithms will enable location, description of defect parameters and a comprehensive assessment of the ceramic tile geometry.

## 2. Materials and Methods 

Three-dimensional vision systems are configured and built strictly with a view of the implementation of inspection and measurement tasks. This requires a detailed analysis of the task in terms of the possibility of determining the parameters describing the tested product. It is necessary to carry out imaging tests both in laboratory conditions and in the conditions of the production line. The 3D vision system presented in this paper was designed and launched by a research team committed to the automation of the product performance inspection process and by specialists involved in the technology of ceramic tile production. Due to the method of three-dimensional image construction being used in the paper, and using the laser triangulation method as part of the preliminary research, the analysis of the cooperation of the image recording system, laser lighting and the examined surface was performed as a function of the adopted configuration of the vision system.

The first stage of the work was to prepare a catalogue of defects found in the products. The task included a description of the defects and characteristic parameters describing each of the defects. In the next stage, a technical solution was developed, enabling three-dimensional imaging of the product surface and defects for the adopted catalogue of defects. Next, three-dimensional image analysis algorithms were developed allowing for a parametric evaluation of each of the defects and marking the defect on the surface of the product.

### 2.1. Characteristics of the Material for Testing

Ceramic tiles with three types of production defects were used for the tests. It was assumed that the product analysis of the ceramic tile surface will be implemented in the scope of checking:

- Microcracking: i.e., cracks visible on irregularly shaped surface, several centimetres long and 100 to 200 μm wide. The morphology of microcracks was characterized by the occurrence of numerous bifurcations in the form of scratches and delaminations visible on the surface of the tiles. Shortcomings of such morphology arise most often as a result of thermal stresses of the second kind that appear during the firing process.
- Tile thickness deformation: with ±1000 μm deviation value, visible in the form of buckling or defects on the surface. This type of production defect is often associated with non-homogeneous distribution of granules filled into the mould before the pressing process.
- Geometrical defects: characterized by non-parallelism of edges and aberration of angles of tile corners with a deviation of up to several degrees from the right angle. This type of defect is related to the occurrence of uneven shrinkage of the tile occurring during the sintering process.

**Figure 1:**
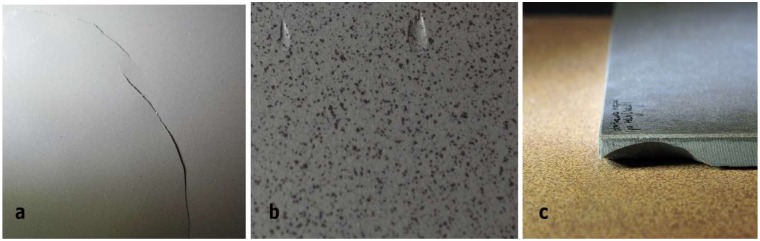
Images of typical production defects of tiles sampled for analysis: (**a**) microcracks with irregular shape, several centimetres long, 100 to 200 μm wide, (**b**) deformation of tile thickness with defects on the surface of the ceramic tile, (**c**) edge defects in the form of material chipping.

Figure 1 presents sample images of typical production defects that are most frequently found in the production process. The above defects are characteristic of ceramic tile production processes and their recognition and limitation in the production process are the basic tasks for the producers of tile ceramics.

### 2.2. Equipment

**Figure 2:**
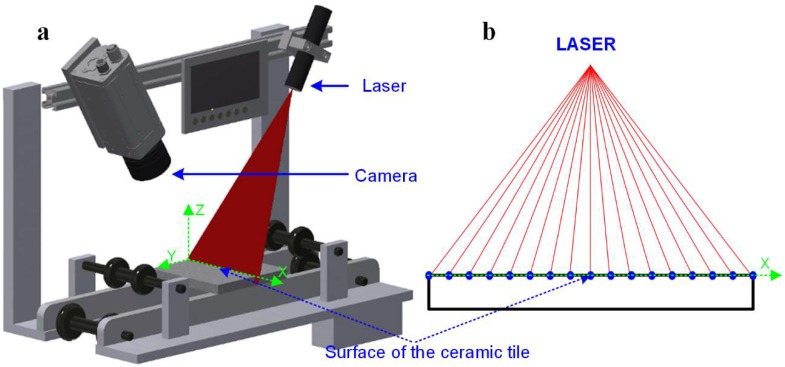
View of the workplace for the detection and assessment of production defects of ceramic tiles: (**a**) the mounting of the camera and laser illuminator relative to the ceramic tile surface, (**b**) the layout of the points describing the height profile on the tile surface in the plane of illumination for the adopted configuration of the 3D vision system.

Figure 2 presents a model of a three-dimensional vision system for detecting and assessing surface defects of tiles using the LTM method [16]. In order to assess production defects of ceramic tiles, the measurement system presented in Figure 2a was used, which includes: 650 nm laser illuminator with a power of 40 mW equipped with an optical system to control the width of the laser line and an additional control system enabling control of the laser power. The Z-Laser type ZM18 laser was used. The work was used a Ranger E55 camera (SICK AG, Waldkirch, Germany) with a matrix with a resolution of 1536 × 512 pixels [16]. 

The vision system works on a control stand designed for the needs of the measuring task. Ceramic tiles are placed on a platform that moves relative to the permanently mounted laser–camera system. The displacement is carried out in the Y-axis of the coordinate system associated with the 3D image. The station propulsion system uses a stepper motor with a propeller with a pitch of 2 mm. The system is controlled from a controller enabling the implementation of a minimum displacement of 0.01 mm. Such displacement is additionally controlled by two encoders. The first is built in the engine axis and the second is mounted on the propeller. This enables displacement control and identification of platform positioning errors.

The geometry of the 3D vision system has been configured for the implementation of the task, including the following parameters: dimensions of the examined “tile” object 192 mm × 192 mm and the required imaging resolution of 0.25 mm in the X- and Y-axes of the coordinate system of the measurement stand. This system allows imaging of 1 mm^2^ at a resolution of 0.25 mm on the plane with 16 measurement points.

### 2.3. Measurement Methodology

The image of the three-dimensional surface of the tile is constructed using surface imaging illuminated with a linear laser beam presented in Figure 2a. Along the laser line, further measuring points are determined enabling the tile height to be described in each of the points (Figure 2b). These points together form a height profile describing the shape of the surface on the X–Z plane. For surfaces without defects, it will be a straight line parallel to the X-axis of the coordinate system. For damaged areas, it will be a curve describing the shape of the surface defect.

Three-D imaging requires determining a set of height profiles that are evenly distributed on the surface of the tile. This is related to the tile displacement relative to the vision system in the direction of the Y-axis. The displacement of the test object relative to the vision system in the determination of successive height profiles is determined as the measurement resolution in the Y-axis of the measurement position. The assembly of a set of height profiles thus determined that are spread evenly on the surface of the tile enables the construction of a three-dimensional image of the surface of the ceramic tile. To calibrate the camera geometry, a built-in model for Ranger cameras was used. Calibration of the power of the laser illuminator and the frequency of the camera’s operation were selected on the basis of statistical tests carried out on prepared sets of tiles manufactured correctly and tiles with damaged surfaces from one production line. Imaging tests were performed for twelve different plates for one plate dimension. Calibration of lighting and system operating frequency should be performed separately for each product group due to changes in the surface structure of the tiles and their colours.

### 2.4. Setting the Height Profile and Determination of Measurement Resolution

**Figure 3:**
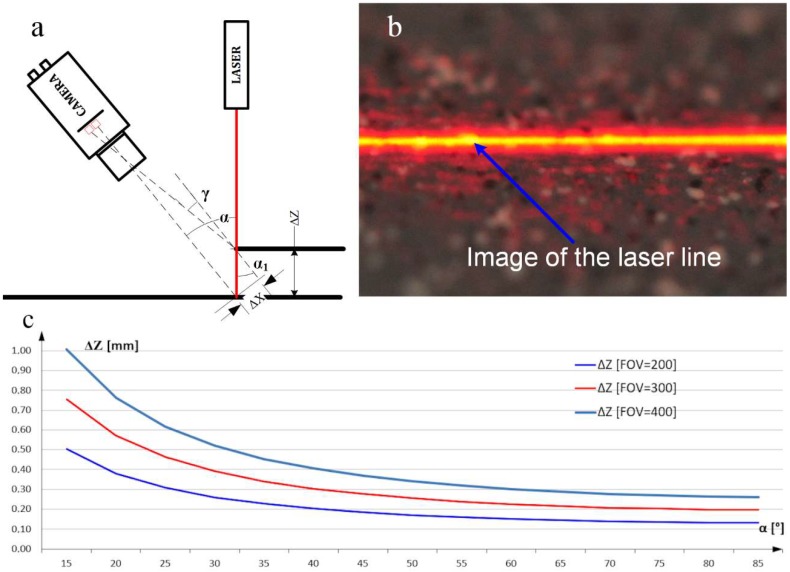
Configuration of the 3D vision system: (**a**) the configuration of the measurement system, (**b**) 2D image of the laser beam illuminating the surface of the tile registered by the camera, (**c**) resolution graph depending for camera angle set for three selected fields of view(FOV).

In the study of the surface of the ceramic tile, the geometry shown in Figure 3a was used, in which the sensor of the vision system is placed at a 90-α angle to the surface of the measuring table. The laser beam is perpendicular to the tested surface and, at the same time, set at the angle *α* to the optical axis of the camera (Figure 3a). The height profile was determined based on the image analysis of the laser line projected on the surface, which is the equivalent of the cross-section in the adopted laser position. The image of the laser line is recorded by the vision system set at the angle of 90-α in the manner presented in Figure 3b. The image recorded by the camera is subjected to analysis that allows determination of a set of points forming the surface height profile. The points that describe the height profile are determined with the resolution that depends on the adopted configuration, the optical system used and the resolution of the matrix of the vision system.

Determination of the resolution of the 3D vision system involves setting three components of the resolution for each axis of the coordinate system associated with the control station. For the Z-axis, this requires determining the minimum height variation of the object described by the ΔZ parameter, at which the laser image is shifted by exactly one row of pixels on the matrix of the vision system (Figure 3a). On the plane parallel to the plane of the matrix, the resolution ΔX in the X axis was determined on the basis of the field of view dimensions (FOV: Field of View) and the resolution of the matrix in pixels. The resolution ΔY in the Y-axis is defined as the displacement of the tested object when determining successive height profiles. Assuming the use of the 768 × 512 pixel matrix area and choosing a video system lens for observation of a 192 mm wide object (FOV = 192 mm), the resolution in the X and Z-axis was determined for the adopted configuration.

∆X = 192 mm/768 pixels = 0.25 [mm/pixel](1)

When calculating the resolution in the Z-axis of the station, the approximation was used assuming that the angle α is equal to the angle *α1*. In fact, the angle *α1* is *α: γ*. Using this simplification, the resolution in the Z-axis is determined for angle *α* = 45°.

ΔZ = ΔX/sin(α) = 0.25/sin(45°) = 0.35 [mm/pixel](2)

where: ΔZ: the resolution in the Z-axis, ΔX: the resolution in the X axis, α: the angle between the optical axis of the laser and the optical axis of the camera.

The resolution in the Y-axis of the coordinate system depends on the shift of the examined area between successive image acquisitions by the vision system. Taking into account the determined resolution specified in the X axis equal to 0.25 mm/pixel, the following values were assumed in the Y-axis:

ΔY = 0.25 mm/pixel(3)

The configuration of the station with the 0.25 mm/pixel resolution in both the X axis and the Y-axis enables description of each square millimetre of the area with 16 measurement points. 

An analysis of the impact of camera angle relative to the laser plane on the resolution possible on a 3D image was performed. The analysis was performed with the same matrix size of 1536 pixels for three FOVs equal to 200 mm, 300 mm and 400 mm. The results of the calculations are presented on the characteristics shown in Figure 3c.

### 2.5. 3D Image Construction

A 3D image built using a 3D vision system is described by a matrix with dimensions (i, j). The dimension j corresponds to the x coordinate in the image coordinate system and defines the width of the field of vision of the video system by specifying the number of columns of the matrix. The dimension i corresponds to the coordinate y in the coordinate system related to the image and defines the number of height profiles determined for the tested object by specifying the number of rows in the matrix. The individual cells of the matrix contain information about the height h (x,y) of the image determined with the resolution ΔZ for each of the points defining the surface of the ceramic tile.

**Figure 4:**
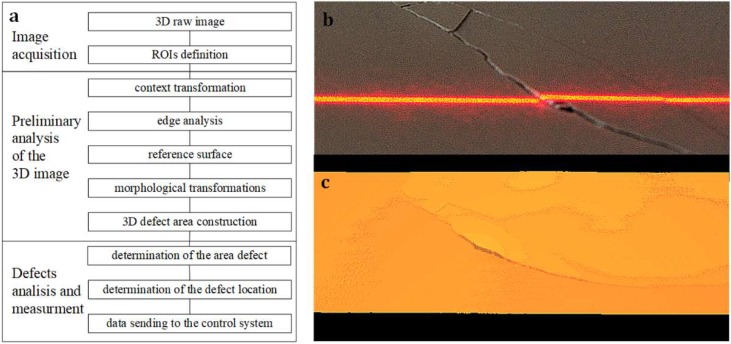
Schematic diagram of the reconstruction measurement algorithm, (**a**) algorithm steps, (**b**) image of the laser line on ceramic surface, (**c**) 3D image of surface.

Composition of profiles enables the construction of a three-dimensional image describing the surface with a resolution of ΔX = ΔY = 0.25 mm in the X and Y-axis and with a resolution ΔZ = 0.35 mm in the Z-axis. Sample images of ceramic tile surfaces made with the adopted resolution are shown in Figure 4b,c. They were produced with a laser source of 20 mW. For every square millimetre of the ceramic tile surface there are 16 measuring points allowing observation of the change in height in selected areas.

The use of 3D imaging and 3D image analysis of the ceramic tile surface may be presented in the form of an algorithm, the diagram of which is in Figure 4a. The 3D image is recorded with a vision system, and then undergoes pre-processing, the purpose of which is to prepare it for the implementation of the measurement procedures. The implementation of the measurement parameters describing the defects makes it possible to locate the fault area and quality classify the product. 

Analysis of three-dimensional images is based on the processing of data describing the height of points forming the surface collected in a two-dimensional matrix. The purpose of this analysis is to determine the characteristic features of the image, e.g., describing surface defects and presenting them in the form of parameters. The most common parameters are: geometrical dimensions, surface areas, volume or shape of defects as defined in the catalogue of defects. Analysis of the image of the three-dimensional surface of ceramic tiles may be divided into three stages: pre-processing, measurements of selected parameters, evaluation of measurement results.

Three-dimensional images were pre-processed to remove distortions and improve the image characteristics that will be used in the phase of measuring the parameters describing the defects [17]. Pre-processing is carried out using point, context and morphological transformations. The point transformations of the image are carried out point by point using the height value of each of the points forming the surface. Three-dimesnional image context transformations allow to transform the value of each point taking into account the value of the points in its vicinity. In the pre-processing phase, morphological transformations are also carried out, allowing, for example, improvement of such image features as edge visibility, edge and surface smoothing. These transformations should be implemented in case of objects with a complex structure and a large number of edges. In case of ceramic tiles, morphological transformations will be used to detect the edges of cracks and microcracks on the surface. As part of the 3D image filtering, a 3 × 3 median filter was used. This protects against single random changes of values on individual pixels. At the same time, a small 3 × 3 mask does not hide cracks of small widths. Additional protection against the lack of information about the height on a single pixel was the use of a Dilate type morphological filter in 3 × 3 mask for surface analysis. For crack detection, the Erode type of morphological filters was used. It allowed artificial enlarging of cracks in a one-pixel width.

## 3. Results

**Figure 5:**
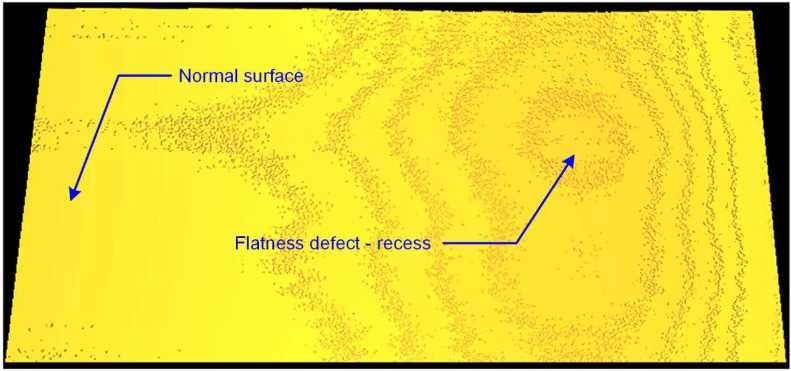
A three-dimensional image of the surface of a ceramic tile with a marked normal area and a defect in the form of a bump.

Figure 5 shows a three-dimensional image of the surface with a flatness defect. The left part of the image shows the flat correct surface of the tile described as “Normal or undamaged surface”. On the right side of the tile, there is a defect in the form of a bump. The parametric evaluation of this defect was made on the basis of the measurement of the deviation between the actual surface of the tile and the reference surface assumed as a perfectly flat pattern.

**Figure 6:**
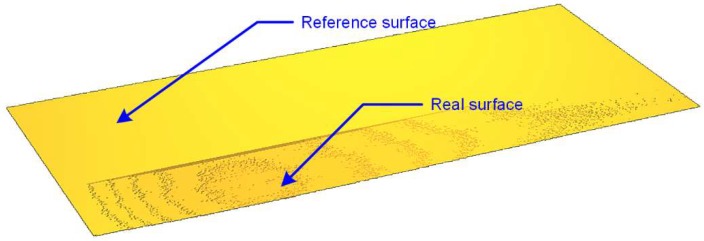
A three-dimensional view of the reference surface describing the correct surface of the tile and the actual surface with the defect.

Figure 6 presents a comparison of the actual and reference surfaces in the area of the defect on the surface of the tile in order to visualize and display the deviation from the ideal surface.

The next step in the implementation of the planar surface flatness algorithm is to determine the surface area of the defect that is outside the tolerance value adopted by the quality control department. 

In order to determine the height deviation in the image, a measurement algorithm is used to determine the values describing the height of the surface along the defined measurement profiles set in the subsequent rows of the image matrix. It is a type of scanning that allows locating areas with the highest flatness deviation and determining the shape of the defect visible on the surface.

**Figure 7:**
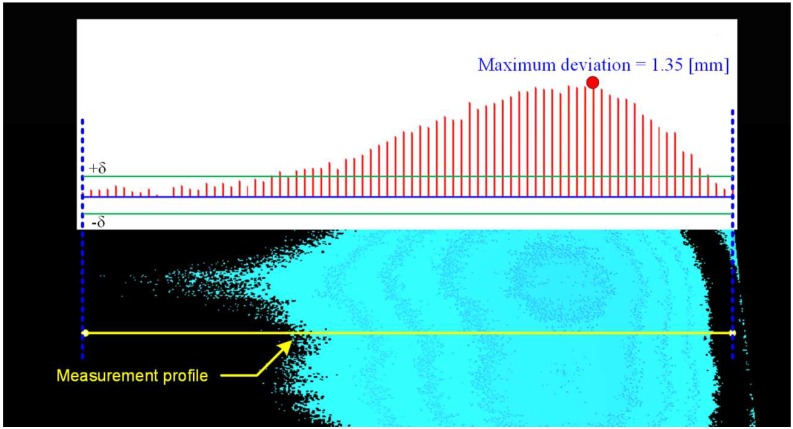
Measurement of the height deviation distribution in relation to the reference plane.

Figure 7 presents a sample measurement profile showing both the value of flatness deviation and the shape of the defect on the surface of the ceramic tile. The analysis is carried out in each row of the image enabling detection of a defect with a field of 1 × 1 px, which corresponds to a field of 0.25 mm × 0.25 mm.

**Figure 8:**
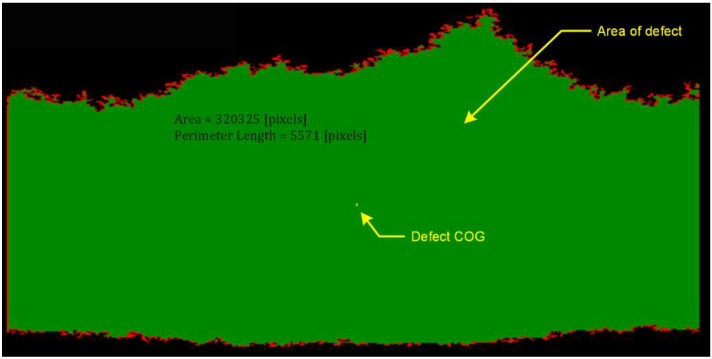
Analysis of shape and measurement of the surface area of the defect.

The shape and area of the area outside the tolerance is determined by comparing the height of each pixel that creates the actual surface image with the height value of the reference plane. It is a point transformation allowing us to determine the surface area of the defect area. An example of the defect area may be found in Figure 8. Image analysis also allows us to determine the edges of the defect, the centre of mass of the defect and the perimeter of the area describing the defect. These features are shown in the figure below as additional parameters describing the defect, and are used in the quantitative assessment of the defect. 

**Figure 9:**
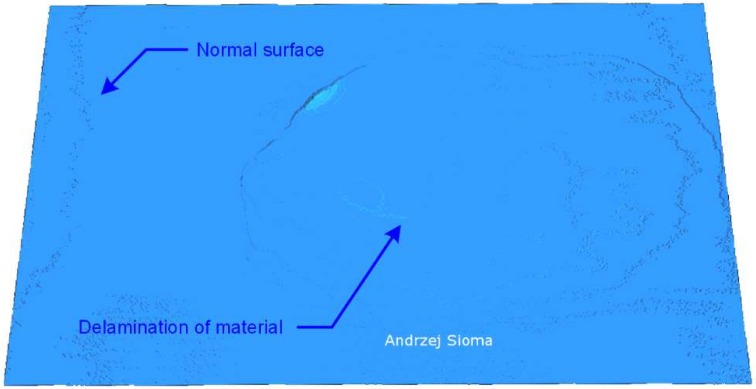
Example of surface flaws in the form of delamination defect on the surface of about 50% of the tile field.

All in all, the algorithm of location and assessment of defect parameters used in the presented example includes the analysis of point-forming surfaces, determination of the reference surface and determination of areas outside the assumed tolerance of surface flatness deviation. This algorithm also allows locating delamination and defects associated with losses and material allowances observed on the surface of the tile. Figure 9 shows the delamination image visible as a separation and elevation of a part of the surface as a result of the stress stored in the ceramic tile material during the pressing and firing operation of the material. The area of delamination is about 50% of the entire tile area.

**Figure 10:**
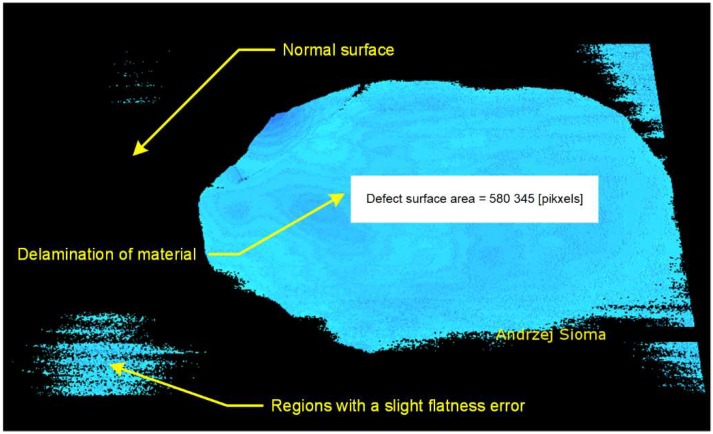
Example of surface flaws in the form of delamination fault on the surface of about 50% of the tile.

The reference plane determined on the basis of the tile surface analysis allows us to indicate the delamination edge and the description of the defect area in the form of delamination. The defect area isolated during the 3D image analysis is presented in Figure 10. In the three-dimensional image, apart from the delamination defect, there is also a defect of parallelism in the form of the raised right edge of the tile relative to the reference plane and a small area in the lower left corner of the tile.

**Figure 11:**
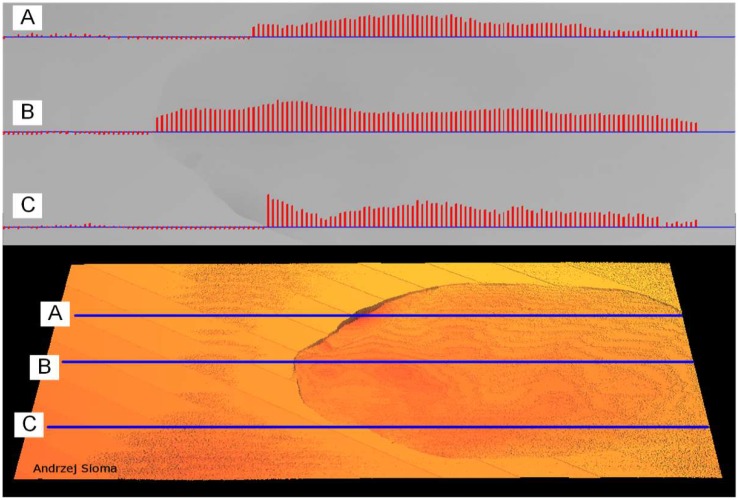
Location of delamination defects and determination of the distribution of height deviation from the reference plane.

The measurement of flatness deviation associated with delamination is also performed by scanning all the image points in the area of the tile. Figure 11 shows three sample height profiles set on the surface. For each profile, the height deviation was measured relative to the reference surface at each point of the profile. Then, for each profile, the distribution of height deviation was plotted. The operation of the analysis of the entire surface of the ceramic tile and the determination of the height deviation for the entire defect area was completed within 200 ms. The area of the defect was determined by the surface area and location of the centre of mass of the defect.

Based on the determined parameters, the defect is assessed and classified. Defects are qualified on the basis of the adopted parameters that describe their acceptable dimensions as prepared by the quality inspection department of the production unit. 

During the research phase, imaging of a set of 50 ceramic tiles selected by the manufacturer with various surface defects was performed. A set of typical defects found in the production of tiles in various dimensions of defects was selected. Each defect was tested in four plate settings, which gave 200 different images of the defects depending on the scan direction of the plate. Subsequent image recordings were made using several sets of imaging parameters: power change, laser mode (continuous pulse) and changes in the frequency profile registration frequency. The set of test images has also been supplemented with tiles surfaces made correctly. The set of images prepared in this way (about 400) was analysed several times with various sets of parameters used in the 3D image evaluation algorithm: changes in the image filtering method, changes in the range of pixels used to determine the reference plane, changes in the method of averaging the pixel height on scanning lines, etc. Preparing the solution in the years 2017–2019, the sets of defects were supplemented, and the algorithm was updated. In addition, tests were carried out in the production line working conditions confirming the effectiveness of the system work.

The biggest threat to the system is the need to shorten the analysis time associated with increasing the efficiency of the production line. This involves the need to reduce the number of analysed measuring points on the 3D surface image. In this case, microcracks with dimensions below 0.25 mm may not be visible.

## 4. Discussion

**Figure 12:**
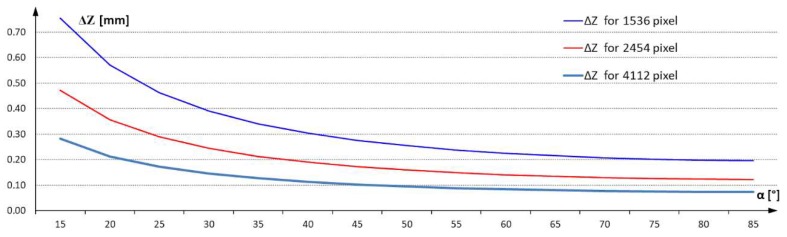
Measurement resolution for selected sensor matrices.

Studies have shown that it is possible to imaging typical defects arising on ceramic tile surfaces. It is also possible to describe the parameters of the defect in the form of its geometric dimensions, surface area and volume. It is also possible to determine the height profile in any cross-section and calculate the deviation of flatness of the surface. All measurements are carried out with the resolution assumed during the construction of the three-dimensional vision system. These are the resolutions determined on each axis of the measuring station. The points describing the surfaces designated by the 3D vision system are subjected to statistical analysis in order to determine the next parameters required by the system user. The article presents a system with resolution dX = dY = 0.25 mm and dZ = 0.35 mm. The measurement resolution can be increased by using correspondingly larger sensor matrices, e.g., Sony IMX304 type with a resolution of 4112 × 3009. Resolution measurements for the presented 3D vision system configuration using selected matrices are shown in the Figure 12 below.

It is possible to make measurements at higher resolutions with a narrow field of view of the vision system and the use of sensor matrices with higher resolution. A system of several vision systems can be used to illustrate the surface of the ceramic tile. However, in industrial conditions, vibrations and other types of noise occur on production lines that prevent imaging with increased resolution.

## 5. Conclusions

The use of three-dimensional image analysis in the field of searching for, locating, and assessing surface defects of ceramic tiles in the form of surface defects such as cracks and delamination allows effective assessment of the quality of the surface of the tile. In addition, the use of a three-dimensional image for fast and industrial surface evaluation in the scope of its geometry allows for integration and extension of control tasks and their implementation at one control station. The presented method of measuring the flatness of ceramic tiles using a three-dimensional image containing 800,000 measurement points allows us to determine a map of the distribution of flatness defects on the tile surface and to check each product within about 200 ms. However, it does not include image building time and communication time with executive devices (machine controllers). It is a significant extension of inspection capabilities in relation to the sensor systems installed on the line enabling measurements at several points on the surface of the tile.

The 3D vision system is designed to perform a specific inspection task. One of the most important parameters is to achieve the required measurement resolution. The presented system enables the registration of a defect image with dimensions of 0.25 × 0.25 mm, which gives 16 measurement points for every square millimetre of the tile surface. This is the limit dimension of the defect that can be observed in a three-dimensional image. If need arises to increase the measurement resolution, the design requirements should be changed, and other components of the system should be selected. The analysis of defect areas on the 3D image allows a repeatable description of the defect parameters and their classification, enabling quick product checking and sorting.

The method of evaluation of ceramic surface defects presented in this paper, based on the 3D image analysis, enables the implementation of a much larger number of measurements and tests, especially in the field of spatial geometric measurements. It significantly extends the possibilities of detailed surface evaluation in relation to analysis using vision systems analysing colours or shades of grey of the tile surfaces. In addition, it significantly expands the detailed microdamage analysis of the surface using 16 measuring points per 1 square millimetre of a ceramic tile surface.
